# Antibody–Drug Conjugates—Evolution and Perspectives

**DOI:** 10.3390/ijms25136969

**Published:** 2024-06-26

**Authors:** Adriana Aurelia Chis, Carmen Maximiliana Dobrea, Anca Maria Arseniu, Adina Frum, Luca-Liviu Rus, Gabriela Cormos, Cecilia Georgescu, Claudiu Morgovan, Anca Butuca, Felicia Gabriela Gligor, Andreea Loredana Vonica-Tincu

**Affiliations:** 1 Faculty of Medicine, “Lucian Blaga” University of Sibiu, 550169 Sibiu, Romania; adriana.chis@ulbsibiu.ro (A.A.C.); adina.frum@ulbsibiu.ro (A.F.); liviu.rus@ulbsibiu.ro (L.-L.R.); gabriela.cormos@ulbsibiu.ro (G.C.); claudiu.morgovan@ulbsibiu.ro (C.M.); anca.butuca@ulbsibiu.ro (A.B.); felicia.gligor@ulbsibiu.ro (F.G.G.); loredana.vonica@ulbsibiu.ro (A.L.V.-T.); 2 Faculty of Agriculture Science, Food Industry and Environmental Protection, “Lucian Blaga” University of Sibiu, 550012 Sibiu, Romania; cecilia.georgescu@ulbsibiu.ro

**Keywords:** antibody–drug conjugates, cancer, targeted therapy, monoclonal antibody, linker, cytotoxic payload, efficacy, safety, clinical trials

## Abstract

Antineoplastic therapy is one of the main research themes of this century. Modern approaches have been implemented to target and heighten the effect of cytostatic drugs on tumors and diminish their general/unspecific toxicity. In this context, antibody–drug conjugates (ADCs) represent a promising and successful strategy. The aim of this review was to assess different aspects regarding ADCs. They were presented from a chemical and a pharmacological perspective and aspects like structure, conjugation and development particularities alongside effects, clinical trials, safety issues and perspectives and challenges for future use of these drugs were discussed. Representative examples include but are not limited to the following main structural components of ADCs: monoclonal antibodies (trastuzumab, brentuximab), linkers (pH-sensitive, reduction-sensitive, peptide-based, phosphate-based, and others), and payloads (doxorubicin, emtansine, ravtansine, calicheamicin). Regarding pharmacotherapy success, the high effectiveness expectation associated with ADC treatment is supported by the large number of ongoing clinical trials. Major aspects such as development strategies are first discussed, advantages and disadvantages, safety and efficacy, offering a retrospective insight on the subject. The second part of the review is prospective, focusing on various plans to overcome the previously identified difficulties.

## 1. Introduction

Even though the first anticancer agents were introduced into therapy in the 1940s, today, around 80 years later, cancer remains a serious disease worldwide. Nowadays, in antineoplastic therapy, there is a constant attempt to approach therapeutic strategies that limit the numerous disadvantages of classical therapy [1,2,3]. Although chemotherapy plays an essential role in treatment strategies for various neoplastic forms, it presents some disadvantages that limit its use, such as lack of selectivity for the tumor cell, inadequate concentration of the drug at the tumor level, the possibility of development for tumor cells resistant to chemotherapy, and the high systemic toxicity of these drugs [4,5].

Advancements in understanding of the cellular and molecular biology of cancer present significant opportunities for the discovery and development of new diagnostic and therapeutic agents for managing this pathology. Lately, innovative antitumor strategies have emerged, utilizing new biotechnologies, nanotechnologies, and targeted molecular therapies as modern approaches to cancer therapy [6,7,8]. 

Among the new therapeutic approaches, monoclonal antibodies and immunotherapeutic drugs have promoted the emergence of novel personalized therapeutic protocols (known as personalized medicine) that have demonstrated remarkable efficacy and minimal toxicity in patients [9].

Antibody–drug conjugates (ADC) consist of a tumor-targeting monoclonal antibody (mAb) conjugated to an active chemotherapeutic molecule via a linker. These ADCs confer selectivity for tumor cells, featuring both the highly specific targeting advantages of mAbs and the extremely potent cytotoxic effect of the chemotherapeutic agent in order to achieve precise and effective destruction of cancer cells [10,11].

**Figure 1:**
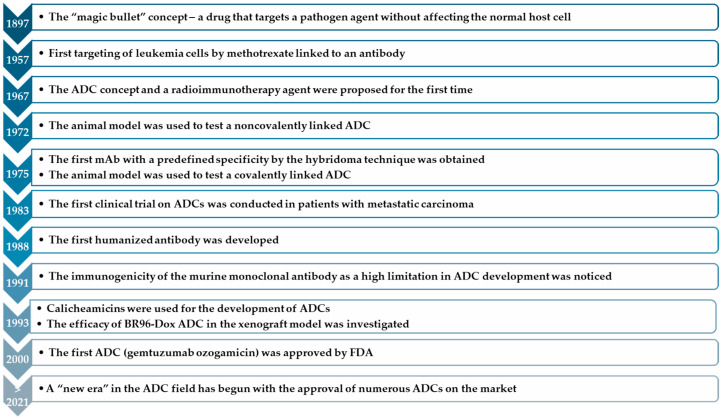
The evolution of ADCs in therapy [13].

The evolution of ADCs in therapy is presented in Figure 1. Thus, the targeted delivery system was first formulated in 1897 by Paul Ehrlich. He conceptualized a manner in which a drug would target pathogen agents while not harming healthy cells. Since that time several advances have been made, like linking an antibody to methotrexate for leukemia targeting cells, proposing the concept of radioimmunotherapy agents and ADCs, and using animal models for testing noncovalent and covalent linkages to ADCs. In 1975, Milstein and Kohler used the hybridoma technique to obtain an mAb with predefined specificity [12,13].

Between 1980 and 1990, the first clinical trials of ADCs for neoplastic diseases were conducted, but the results were not satisfying due to increased toxicity and rather limited efficacy [12]. An example is a phase I clinical trial of a chimeric anti-Lewis Y (Le(Y)) monoclonal antibody conjugated to doxorubicin, BR96-Doxorubicin (BR96-Dox), administered to patients with tumors that expressed the Le(Y) antigen [14]. Also, the first humanized antibody was developed, the immunogenicity of the murine monoclonal antibody as a high limitation in ADC development was noticed, calicheamicins were used for ADC development because of their increased cytotoxicity, and the efficacy of BR96-Dox ADC in the xenograft model was investigated (Figure 1) [12].

Later, in 2000, the beginning of the directed and targeted strategy of ADC drugs in antineoplastic therapy was marked after the Food and Drug Administration (FDA) approval of first ADC drug, gemtuzumab ozogamicin (GO), for use in adult patients with acute myeloid leukemia [12]. In 2010, the drug was withdrawn from the market, but later it was relaunched (in US—2017, EU—2018) [15,16,17]. After about a decade, in 2011, brentuximab vedotin (BV) was approved for the treatment of Hodgkin lymphoma and systemic anaplastic large cell lymphoma, followed by adotrastuzumab emtansine (T-DM1), approved in 2013 for solid tumors (breast cancer). The latter shows greater specificity on the human epidermal growth factor receptor 2 (HER2) than the others, being at the same time the first ADC that could be used on solid tumors [12,18,19].

Until December 2021, a number of 14 ADCs were approved worldwide by the FDA and the European Medicines Agency (EMA) for both hematological malignancies and solid tumors [10]. Of these, T-DM1, enfortumab vedotin, fam-trastuzumab-deruxtecan, and sacituzumab govitecan are approved for the treatment of solid tumors, while the other agents are indicated for hematological malignancies. The strategies applied to increase the efficiency of these compounds aim at the most accurate dosing for better tumor penetration and thus minimizing toxicity in order to offer the possibility of expanding the therapeutic benefits of ADCs [20]. Currently, more than 100 ADC compounds are in different phases of clinical trials, aiming to gradually replace conventional chemotherapy [21].

In contrast to conventional cytotoxic drugs, ADCs present numerous advantages, thus combining the targeting benefits and pharmacokinetic characteristics of the mAb with the high capacity of killing cancer cells of the cytotoxic payload. In this manner, the toxicity associated with drugs with a narrow therapeutic index is managed without affecting healthy cells [22]. Unlike conventional chemotherapy, which generally lacks selectivity for cancer cells, ADCs target certain types of cancer cells by internalizing the ADC–antigen complex by endocytosis, after which it is directed to the target site of action to be disassembled, releasing the payload so as to selectively destroy the cancer cell, minimizing off-target cytotoxic effects [23]. 

However, several factors contributing to the toxicity of ADCs limit their selectivity for cancer cells: (1) the instability of the linker-drug bond that can cause the release of the payload into the circulation prematurely (payload-dependent toxicity); (2) if the target antigen required for mAb coupling is expressed in non-malignant cells, this may affect the distribution of the cytotoxic drug and the place of accumulation, resulting in toxicity that is independent of the payload; (3) the binding of ADCs to the fragment crystallizable region (Fc) of the antibody receptors (FcγRs, FcRn and C-type lectin) which facilitates their uptake into non-malignant cells [23].

The aim of this review was to provide information regarding ADCs from multiple perspectives. Aspects like structure, conjugation, and development particularities alongside effects, clinical trials, safety issues and perspectives, and challenges for future use of these drugs were discussed. As many reviews regarding drug conjugates were published, we aimed to combine chemical aspects of ADCs with pharmacological aspects, clinical considerations, and safety issues of these drugs, thus providing the readers with a better understanding of this subject. The information provided in this narrative literature review could be used for future studies.

## 2. Structural Aspects of ADCs

**Figure 2:**
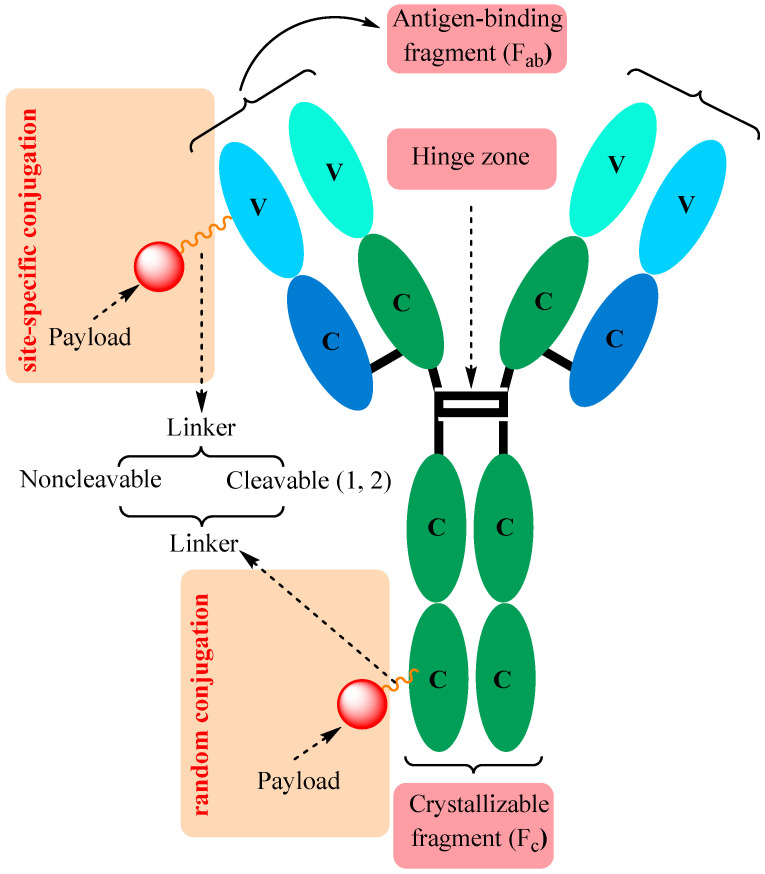
Main components of (ADCs); 1—chemically cleavable linker; 2—enzymatically cleavable linker. Adapted from [11,25,26,27].

As presented in Figure 2, the main components of an ADC are three basic elements: monoclonal antibody, linker, and payload [11,24,25,26,27].

### 2.1. Monoclonal Antibodies

All antibodies have a shared basic structure (Figure 2) with two heavy polypeptide chains (green) and two light chains (blue), both of which are composed of different regions that are either constant (C) or variable (V) in sequence. They are assembled into a Y-shaped structure by means of a number of inter- and intrachain disulfide bonds and also different non-covalent interactions. Short carbohydrate chains that are bound to heavy polypeptide chains enhance the water solubility of the mAb, and a flexible “hinge” region located in the middle of the antibody enables it to adapt to various arrangements of antigens on target cell surfaces [11,25,26,27,28].

The mAb is the main component of the ADC structure [24]. It is necessary for the specific attachment between the target antigens and the ADC, specific for an antigen predominantly expressed on a tumor cell with the role of transporting the payload (cytotoxic drug) to the targeted site of action [10]. To maximize ADC efficacy, selection of an antibody must prioritize a well-characterized antigen with minimal or absent expression in normal tissues while minimizing off-target toxicity [23]. In the initial phases of ADC drug development, antibodies obtained from laboratory animals (mice) were used. However, this approach resulted in high failure rates due to severe side effects caused by immunogenicity [10]. In the last decades, with the development of recombinant DNA technology, murine antibodies have mostly been substituted with chimeric, humanized or human (fully humanized) antibodies, the latter showing a significant reduction in immunogenicity [29]. Another important aspect is the internalization efficiency of the ADC–antigen complex, which depends on the binding affinity of the antibody to the antigen. Adequate affinity is essential for the rapid internalization of the ADC–antigen complex. Very frequently used mAbs in ADCs are the human IgG isotypes, which have the advantage of a long half life, the capacity to bring about heightened antibody-dependent cell-mediated cytotoxicity and complement-dependent cytotoxicity against cancer cells [23,30]. IgG1 is the generally used subtype for ADCs, the most common in serum, with important effector functions like antibody-dependent cell-mediated cytotoxicity, antibody-dependent phagocytosis, and complement-dependent cytotoxicity through an increased attachment affinity with the Fc receptor. IgG3 is less often included in ADCs due to its rapid clearance rate. In general, the high molecular weight of IgG antibodies (approximately 150 kDa) frequently poses a challenge for diffusion through blood capillaries and in tumor tissues. That is why for the design of ADCs, especially for solid tumors, the size of the antibodies was intended to be reduced by eliminating the Fc segment [10].

### 2.2. Linkers

Linkage groups that bind the cytotoxic agent to the antibody aim to be stable in circulation and deliver the cytotoxic agent within the target cells. The linker has a crucial part in the delivery of the cytotoxic active molecule at the level of the neoplastic tissue, in the cancer cell [23].

In order to contribute to the structural organization of a selective and powerful ADC, the linker must have the following essential characteristics: (i) have an appropriate stability for a long time in the blood stream in order to prevent early release of the cytotoxic drug and off-target effects while simultaneously enabling effective delivery of the payload into the target cancer cell; (ii) the linker must assure a good solubility of the ADC, for bioconjugation; (iii) the attachment specificity of the antibody should be uncompromised by the binding of the linker to the mAb.

The conjugation site and selection of the linker are essential for the stability and pharmacokinetics of ADCs, and the stoichiometry of the payloads given by the linker dictates their homogeneity and stability [23]. Mainly, linkers are classified into two categories taking into account the stability of the connection with mAb in the systemic circulation and depending on the mechanism of targeted release: cleavable linkers and non-cleavable linkers (Table 1). These linkers have a major importance in the pharmacokinetic characteristics, the selectivity, the therapeutic index, and in general the efficiency of the ADC. In the evolution and research of ADCs, various linkers have been approached. Cleavable and non-cleavable linkers have been shown to be safe in preclinical and clinical studies. Linkers can also be classified based on their mechanism of drug release and their stability in the blood stream [26].

#### 2.2.1. Cleavable Linkers

Cleavable linkers release the drug into the target cell in response to the physiological environment [23]. They are labile structures cleavable depending on some intracellular circumstances such as acidic pH, reduction–oxidation reactions, predominant expression in glutathione, or expression of relevant enzymes, especially the action of lysosomal proteases. In the tumor microenvironment with acidic pH, they can penetrate in nearby tumor cells without expression of the targeted antigen, inducing the bystander effect [31]. The cleavable linkers are divided into several groups.

##### pH-Sensitive Linkers

pH-sensitive linkers are cleavable linkers with an acidic group, like a hydrazone group, which releases the drug at the low pH value of the lysosome [23]. This strategy is based on the use of an environment with a lower pH of the endosome (pH = 5–6) and lysosome (pH = 4.8) compartments as opposed to the cytoplasm (pH = 7.4) [32].

**Figure 3:**
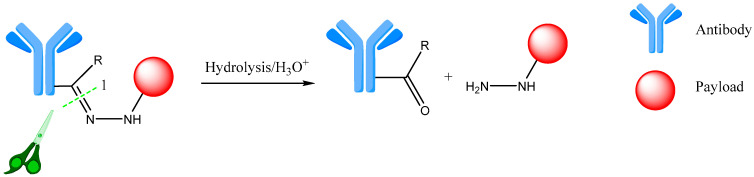
Hydrazone linker cleavage. 1—site of hydrolysis. Adapted from [31,33].

A generalized example of this type of linker is presented in Figure 3 [31,33].

**Figure 4:**
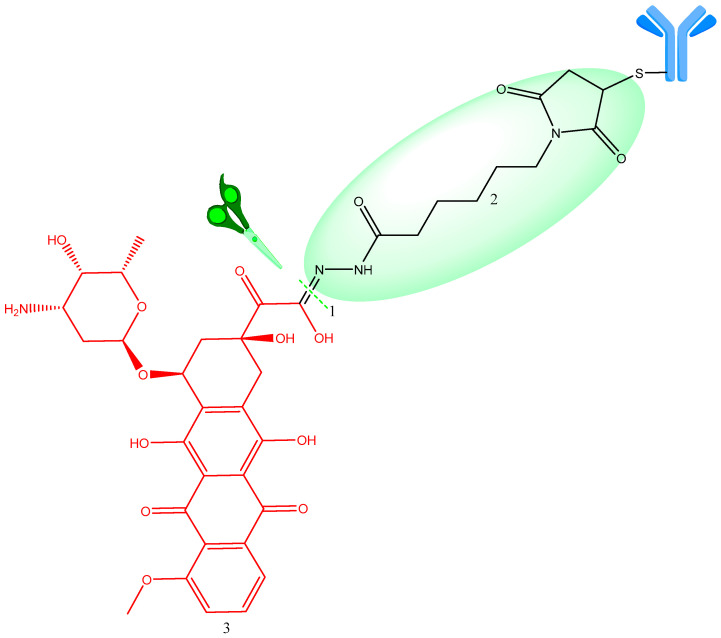
BR96—Doxorubicin ADC structure. 1—Site of hydrolysis; 2—(6-maleimidocaproyl) hydrazone linker (highlighted in green); 3—payload (doxorubicin) (red). Adapted from [26].

A relevant example for this type of linkers is R96-Dox in which the active cytotoxic doxorubicin (DOX) molecule is conjugated with an acid-sensitive linker, which blocks DNA replication, a hydrazone linker (6-maleimidocaproyl) linked to the cysteine (Cys) residues of the antibody humanized monoclonal BR96 (Figure 4) [26].

Sometimes this type of linker can be characterized by instability under physiological conditions, which limits its usefulness [34]. For example, regarding the inotuzumab ozogamicin product approved in 2017, in vivo hydrolysis of hydrazone has been shown to occur at a rate of 1.5–2% per day over 4 days. This ADC with the active metabolite calicheamycin contains a recombinant humanized anti-CD22 antibody, linked to N-acetyl-γ-calicheamicin dimethyl hydrazide through the acid-labile hydrazone linker (4-(4′-acetylphenoxy)butanoic acid) [26].

##### Reduction-Sensitive Linkers

Reduction-sensitive linkers include a disulfide bond that is susceptible to reduction by glutathione, exploiting the elevated intracellular glutathione levels present in cancer cells [23]. Also, they represent an alternative to the more unstable hydrazone linker under physiological conditions. The disulfide bond is encapsulated within the linker, resisting reductive cleavage in the blood flow [35]. Optimization of the steric obstacle of disulfide bridges may decrease premature drug release [11]. 

**Figure 5:**
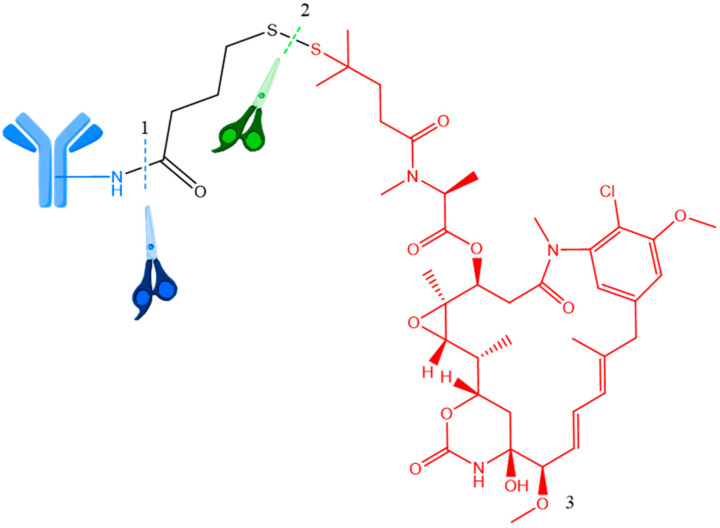
Cantuzumab ravtansine (huC242-SPDB-DM4) conjugate cleavage. 1—Site of first cleavage (proteolytic); 2—site of second cleavage (reduction of disulfide bond by cytoplasmatic glutathione); 3—payload (ravtansine, red). Adapted from [35,36,37].

The main premise for cleavable ADCs with these linkers is the difference in the reduction potential within the intracellular environment. Glutathione, which is highly expressed and released during cell replication, is present in significant concentrations in cancer cells. Reducible linkers produce a neutral payload that is able to diffuse into adjacent cancer cells, and thus the bystander effect occurs [26]. Cantuzumab ravtansine (huC242-SPDB-DM4) has a disulfide linker ADC featuring a disulfide linker, a tubulin-targeting huC242 antibody, and an active cytotoxic molecule (Figure 5). In this process, the ADC undergoes proteolytic cleavage, followed by the breakdown of the disulfide bond to release the active drug, which is then enzymatically metabolized by S-methyltransferase [26]. Thus, both the structure and release mode of the cytotoxic active molecule are presented [35,36,37]. 

In vitro and in vivo studies were conducted to assess the efficacy, pharmacokinetics, and toxicity of trastuzumab–maytansinoid conjugates (microtubule depolymerizing agents) employing both disulfide and thioether linkers. It was thus demonstrated that the non-reducible thioether bond showed enhanced activity in comparison to unconjugated trastuzumab or trastuzumab bound to other maytansinoids by disulfide linkers [38].

GO is an example involving a hydrazone linker as well as a disulfide bond. To prevent the premature release of the cytotoxic active molecule of calicheamycin and to increase the stability of this bond, two methyl groups were inserted into the carbon α which carries the disulfide bonds. Under strong acidic conditions, linker hydrolysis occurs, when the active metabolite—calicheamycin—is initially released through hydrolysis of the hydrazone fragment, succeeded by cytosolic reduction of the disulfide bond to produce the free sulfide anion. Later, it produces a thiophene ring by cyclization [26].

##### Phosphate-Based Linkers

Phosphate-based linkers can significantly improve linker hydrophilia, and although the exact hydrolysis mechanism has not been confirmed when using these linkers, the phosphate/pyrophosphate structure is a promising new linker for obtaining ADCs. Thus, the traditional cathepsin B-sensitive Val-Cit-PAB linker was replaced with a phosphate diester structure and linkers based on the structure of monophosphate, pyrophosphate, and triphosphate diesters were synthesized. 

**Figure 6:**
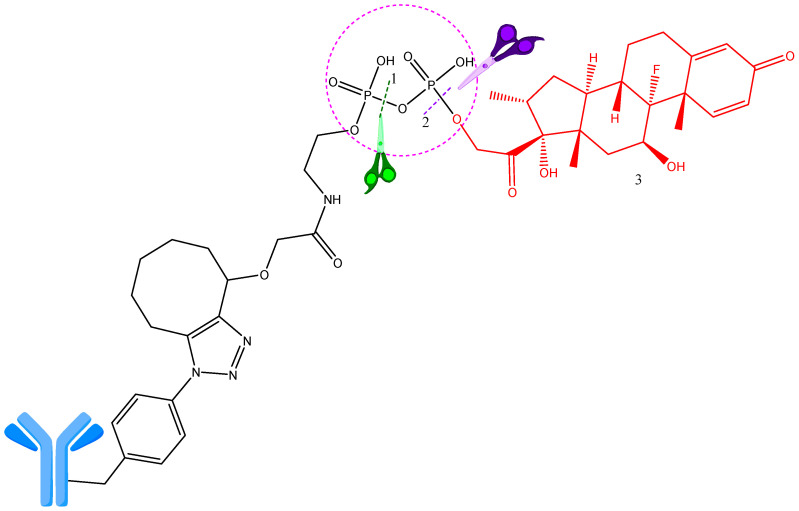
Pyrophosphodiester linker cleavage in lysosome by pyrophosphatase and phosphatase. 1—Site of pyrophosphate cleavage (by pyrophosphatase); 2—site of phosphate cleavage (by phosphatase); 3—payload (dexamethasone) (red). Adapted from [31,39].

In vitro studies show that ADCs containing hydrophilic linkers of pyrophosphate and triphosphate diester (Figure 6) have important advantages. They are cleaved faster than monophosphate diesters. When metabolizing pyrophosphate diesters, ADCs are initially hydrolyzed to a payload monophosphate metabolite, which then rapidly releases the active molecule [31,39].

The anionic linker phosphate/pyrophosphate shows a higher solubility in water than traditional linkers and at the same time has a very good stability. Moreover, following internalization, pyrophosphodiester is swiftly cleaved via the endosome––lysosome pathway, delivering the unchanged active molecule. A particular pyrophosphatase-based linker employed in anti-CD70 ADCs has also been developed. It releases payloads containing hydroxyl, dexamethasone, and fluticasone propionate [40]. 

##### Peptide-Based Linkers

**Figure 7:**
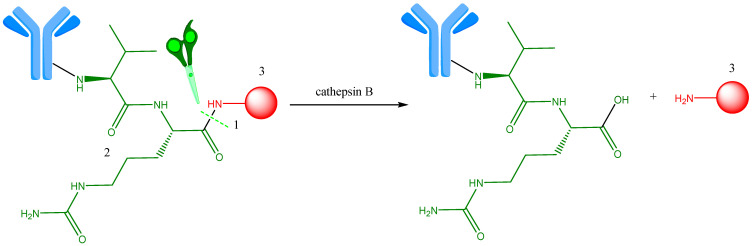
Val-Cit linker enzymatic cleavage by cathepsin B in lysosome. 1—Site of enzymatic cleavage; 2—Val-Cit linker (green); 3—payload (red). Adapted from [41].

The role of peptide-based linkers is to keep ADCs unchanged in the systemic circulation and facilitate the release of the cytotoxic molecule through cleavage by specific intracellular proteases, like cathepsin B [35]. Schematically, this cleavage is represented in Figure 7 (a proteolysis reaction and cleavage sieve in lysosomes) [31,41].

Cathepsin B is a lysosomal protease enzyme, overexpressed in different cancer cells, with a rather large range of substrates, but it exhibits a preference for recognizing the phenylalanine–lysine (Phe-Lys) and valine–citrulline (Val-Cit) sequences. The C-terminal end of these sequences splits the peptide bond. Val-Cit and valine–alanine (Val-Ala) linkers coupled with p-aminobenzyloxycarbonyl (Val-Cit-PABC and Val-Ala-PABC) are ones of the best cleavable linkers for ADC [35,42]. Due to inappropriate pH levels and the presence of serum protease inhibitors, these peptide linkers, such as the dipeptide linker Val-Cit or Phe-Lys, exhibit enhanced systemic stability while facilitating rapid enzymatic drug release into the target cell. This linker, used in many ADCs, has the advantage of keeping the stability of ADCs in plasma and favoring cleavage under the action of intracellular protease [35]. Thus, BV is an example of ADC in which the cytotoxic active molecule is conjugated to an anti-CD30 antibody by an auto-immolative (cathepsin B-sensitive PABC) linker. While ADCs that contain this linker are typically stable under physiological conditions, an unknown serine protease enzyme has been found to cleave the linker in experiments on laboratory animals [26,43].

##### β-Glucoronide-Based Linkers

**Figure 8:**
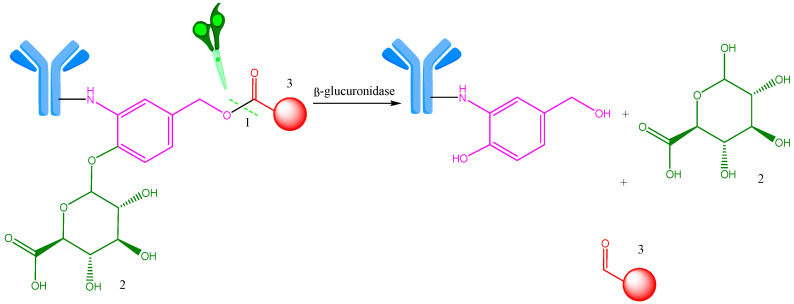
β-Glucuronide linker enzymatic cleavage by β-glucuronidase in lysosome. 1—Site of enzymatic cleavage; 2—β-glucoronide-based linker (green); 3—payload (red). Adapted from [26].

β-glucuronidases are hydrolytic lysosomal enzymes located exclusively in the lysosomal compartment of the cell and they are enzymatically active in the extracellular environment. These enzymes degrade the β-glucuronic acid molecule into polysaccharides and are active in hydrophilic media to release payloads from conjugates [26,35]. Schematically, this cleavage is shown in Figure 8.

The introduction of auto-immolative groups improves linker stability and facilitates the safe release of the cytotoxic active molecule [35]. Linker β-glucuronide has been used to conjugate multiple mAbs in a number of ADCs for various cytotoxic drugs, including auristatin derivatives monomethyl auristin E and F and propyloxazoline doxorubicin [35,44,45]. In addition to utilizing auristatins and doxorubicin, this approach has also been used to target special classes of cytotoxic agents like anthracyclines, camptothecin derivatives, taxanes, mustard nitrogen derivatives, and histone deacetylase inhibitors [35]. 

#### 2.2.2. Non-Cleavable Linkers

**Figure 9:**
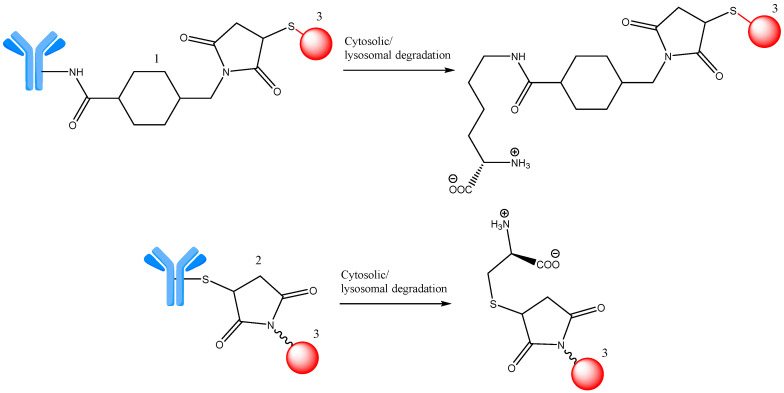
Cytosolic/lysosomal degradation of ADCs with non-cleavable linkers. 1—MC; 2—thioether linker; 3—payload (red). Adapted from [46,47].

Structurally, non-cleavable linkers are of two types, namely thioether or maleimidocaproyl (MC). These linkers make stable bonds that inhibit proteolytic cleavage and provide increased stability in plasma compared to their cleavable counterparts. ADCs incorporating this type of linker rely on the full lysosomal enzyme degradation of the antibody for post-internalization payload release, leading to concomitant linker detachment [35]. ADCs with non-cleavable linkers first require the ADC to be internalized and mAb to undergo degradation by lysosomal proteases in order to deliver the active molecule (Figure 9) [40,46,47]. Therefore, when designing an ADC, the option of a non-cleavable linker instead of a cleavable one is a decision for a particular strategy in which the stability of the complex is more important than the rapid release of the active fragment. These linkers can have different lengths, polarities, favorable stability, and flexibility so they can be used for different types of ADCs [48]. 

**Figure 10:**
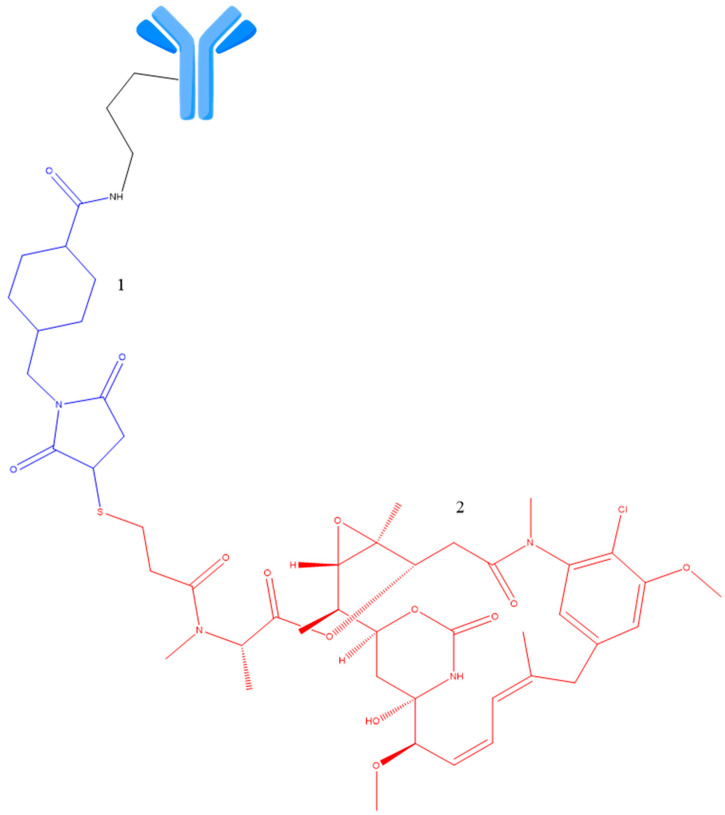
Structure of Trastuzumab emtansine. 1—Non-cleavable SMCC linker (blue); 2—payload (emtansine, red). Adapted from [49,50].

Several non-cleavable linkers have been studied for ADC research, the typical one being N-succinimidyl-4-(N-maleimidomethyl) cyclohexane-1-carboxylate, existing in trastuzumab emtansine, which is conjugated to the cytotoxic active molecule to lysine (Lys) residues of the anti-HER2 trastuzumab mAb (Figure 10) [49,50]. Catabolism of such constructs resulted in Lys-SMC-DM1 as a major tumor metabolite [40,51]. Additionally, drugs connected to such linkers typically cannot carry out the bystander effect because released catabolites have reduced cell permeability [52]. 

Compared to cleavable linkers, the primary benefit of non-cleavable linkers is represented by improved plasma stability, potentially resulting in a wider therapeutic window. Furthermore, it is predicted to decrease off-target toxicity because inseparable ADCs have greater stability and tolerance [40,46]. Numerous studies have been conducted to design and select appropriate linkers to obtain ADCs by conjugation with monoclonal antibodies and cytotoxic drugs, which are the components that can alter the stability, toxicity, pharmacokinetic properties, and pharmacodynamics of ADCs. Every linker has its benefits and shortcomings, and therefore numerous aspects must be taken into account in their choice and use. Thus, the decision to select the suitable linker considers existing groups in mAb, reactive groups in the cytotoxic medicinal product, and derived functional groups. The optimal linker ensures sufficient stability of the cytotoxic drug in the blood, efficiently inhibits early release of the drug, and efficiently facilitates the release of the cytotoxic drug into targeted tumor cells, thus enhancing the efficiency and tolerability of an ADC in its ensemble [31,53,54].

### 2.3. Payloads 

Payloads are cytotoxic active molecules coupled with an mAb via a linker [55]. Over time, numerous molecules such as cytokines [56], radionucleotides [57], and various toxins [57] have been suggested as payloads, but currently, most FDA/EMA-approved ADCs are conjugated with small cytotoxic molecules with a wide array of structures and mechanisms of action [52,58,59]. ADCs release payloads into the intracellular environment after lysosomal cleavage or as a result of a modification in the environment such as the redox potential. The high potency of the cytotoxic active molecule is of utmost importance, as it is closely related to the high selectivity to the target tissue. Payloads bind to targets, such as microtubules or genomic DNA, thereby inhibiting tumor cell proliferation [60,61,62]. 

Payloads in ADCs can be categorized into two main classes: microtubule inhibitors (maytansinoids or auristatins) and DNA-affecting agents (topoisomerase inhibitors or inter travelers DNA) [52,60]. The payloads in ADCs are required to meet several important requirements [52]:

(i) high cytotoxic capacity correlated with the accentuated lipophilic character;
(ii) the target of the payload has to be situated inside the cells;
(iii) the active cytotoxic molecule should be small in size, should not have immunogenicity, and should have adequate solubility in aqueous buffer solutions so that conjugation takes place in the best possible conditions;
(iv) the payload has to be stable in plasma.

**Figure 11:**
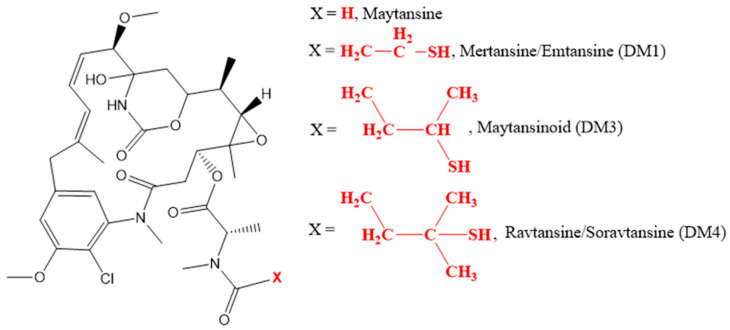
Chemical structures of maytansinoids. Adapted from [63,64].

Maytansinoids (Figure 11) like mertansine are synthetic derivatives of maytansine, an inhibitor of microtubule polymerization. There are two derivatives of maytansine: DM1 and DM4. DM1 includes emtansine and mertansine and DM4 includes soravtansine and ravtansine [63,64]. It binds to tubulin, resulting in mitotic arrest and cell death [52,65]. The trastuzumab–emtansine conjugate DM1 (T-DM1) is the first ADC approved for treating solid tumors [66]. 

Auristatins are synthetic compounds derived from a natural antimitotic drug, dolastatin 10, which is isolated from a marine gastropod mollusk, *Dolabella auricularia*. Monomethyl auristatin E (MMAE) is an exceptionally potent antimitotic agent which prevents cell multiplication by inhibiting tubulin polymerization. Also, monomethyl auristatin F is a new auristatin derivative with a loaded C-terminal phenylalanine that diminishes its cytotoxic activity as opposed to its unloaded correspondent, MMAE [58,67]. The inhibition of microtubule polymerization by targeting tubulins leads to stopping and apoptosis in the second growth phase/mitosis (G2/M) cell division phase [68].

Other payloads are cytotoxic molecules that act as topoisomerase I inhibitors, which increase the antitumor immune response. The following are examples of ADCs with payloads that act as topoisomerase I inhibitors: sacituzumab govitecan and trastuzumab deruxtecan [13,69]. The agents acting on DNA are calicheamycin, pyrrolobenzodiazepine dimer (PBD), indolinobenzodiazepines, duocarmycin, and doxorubicin. For example, dimers of duocarmycins and PBDs bring about DNA alkylation by binding alkyl radicals to guanine-rich areas, calicheamycins, and bring about double-strand DNA breaks, leading to cellular apoptosis [70,71,72].

## 3. Antibody–Drug Conjugation and Efficiency

Antibody conjugation and cytotoxic payload can affect the pharmacokinetics and therapeutic index of ADCs. Traditional drug conjugation typically takes place on the mAb backbone, either through alkylation or acylation of lysine side chains, as employed in GO and trastuzumab emtansine, or through reduction in disulfide bonds which can release Cys residues to be bound to linkers as used in BV [24]. Traditional drug conjugation strategies in ADCs are random, leading to a heterogeneous mixture of ADCs and to diverse pharmacokinetic profiles, efficacy and safety [73]. To date, all approved ADCs are obtained by coupling reactions by non-specific modification of Cys or Lys antibody residues, or by using reduced disulfide structural linkers, inevitably resulting in heterogeneous conjugates with limited therapeutic efficacy. 

The conjugation process through Cys residue results in the partial reduction in four interchain disulfide bonds of antibodies, generating as many as eight reactive thiol groups. The partially reduced antibody is then conjugated to a payload that contains a maleimide linker with thiol groups. For instance, in the BV used in Hodgkin lymphoma, the payload employed is MMAE containing the protease-cleavable maleimide linker. While BV exhibits lesser heterogeneity compared to T-DM1, it comprises numerous ADC molecules containing zero to eight payloads [74].

**Figure 12:**
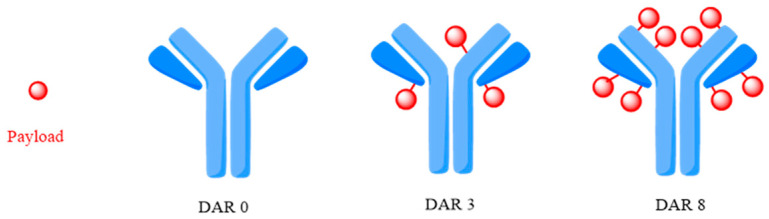
Drug to Antibody Ratio (DAR) illustration. Adapted from [76].

An essential factor in evaluating the efficiency of an ADC is the Drug–Antibody Ratio (DAR) (Figure 12), defined as the number of drug molecules bound to an mAb. An optimal DAR depends on the nature of the payload [52,75,76]. DAR varies greatly and depends on other ADC variables contingent on the conjugation site and the use of light or heavy conjugated chains. The DAR value influences drug efficacy due to decreased potency resulting from low drug loading, while increased drug loading can affect toxicity and pharmacokinetics (PK) [77,78]. Currently, the DAR value of approved ADCs is 2–8. A PK optimal DAR value of four has been questioned by the approval of some recent ADCs with a DAR of eight, particularly due to enhanced hydrophobicity masking technologies. The technology that conjugates the mAb to the linker by positioning the active molecule is critical to achieving a homogeneous and controlled DAR [72].

In general, ADCs with increased DAR are more active in vitro and are more rapidly distributed to plasma. For instance, in vitro, the biological activity of BV depends on the DAR value. In preclinical studies conducted on laboratory mice, the ADC with a DAR 8 exhibited plasma clearance five times faster than the version with a DAR 2, yet it did not demonstrate any enhancement in cytotoxic activity [72]. This behavior is closely related to the increase in the hydrophobic character of the antibody–linker complex that can be bypassed by using hydrophilic structures that do not affect plasma clearance (e.g., for sacituzumab govitecan (SG), a higher DAR is associated with higher antitumor activity in vivo) [21]. SG results from the conjugation of an anti-Trop-2 (hRS7) IgG antibody with SN-38 (the active metabolite of irinotecan) through a CL2A linker fragment. The internalizing antibody IgGκ RS7–3G11 (RS7) was initially developed in mice to bind Trop-2 with nanomolar affinity and was later replaced by humanized mAb for clinical use [79]. 

Based on the used conjugation method, a mixture of ADC species with different DARs and binding sites can be produced. Target-specific conjugation methods provide a more homogeneous product with defined DAR using modified residues, modified glycans, enzymatic linkages, and chemical crosslinkers. Cys and Lys binding modes generally lead to heterogeneous mixtures in the DAR [58,80]. Notwithstanding the conjugated amino acid (Cys or Lys), the obtained ADCs are heterogeneous products with DARs exhibiting a Gaussian-like distribution, which highlights two potential concerns: (i) a restricted quantity of unconjugated antibody may remain in the product, thereby attaching and filling ADC attachment sites and diminishing effectiveness; (ii) ADCs with higher DARs are more active in vitro, not as well tolerated in vivo, and exhibit swifter clearance as opposed to lower DAR values. 

Two kinds of conjugation exist: stochastic conjugation and site-specific conjugation (SSC). In general, stochastic conjugates predominate, but the functional and analytical benefits of site-specific conjugates are highlighted [81]. To avoid these difficulties, SSC methods allow the synthesis of homogeneous ADCs, optimizing both the position for conjugation, with enhanced biological activities, and their analytical characterization [81]. SSC on Cys is the method in which mAbs designed with Cys residues and interchain disulfides allows the performance of maleimide conjugations for the exploitation of existing payloads [82]. A bioconjugation procedure is usually applied to attach the mAb to a linker and payload. Overall, the conjugation process is of utmost importance for every technology involved in obtaining an ADC. This step determines the nature and characteristics of the bioconjugate and significantly influences the conjugation efficiency. An effective conjugation strategy greatly contributes to the success of any ADC [83]. Typical conjugation approaches are random conjugation to Lys residues and conjugation to reduced Cys residues in the hinge region of mAbs, with more recently developed techniques such as SSC which is a major preoccupation in recent years [46,84]. Such approaches encompass the introduction of modified Cys residues or unnatural amino acids into the antibody sequence or enzymatic conjugation by transglutaminases and glycotransferases [73]. Although the DAR profile can be regulated by specific conjugation techniques, SSC methods can lead to more homogeneous drug products that can enhance yield and biophysical properties. Protein engineering approaches have enabled the strategic placement of residues in specific locations, allowing chemo-selective conjugation reactions. Thus, conjugation stability depends on specific modified Cys sites able to enhance the therapeutic index, with the concomitant increase in conjugation stability and PK characteristics [58].

In Table 2, the methods used to optimize the effectiveness of some ADCs using different strategies aimed at DAR are schematized [75,85].

**Figure 13:**
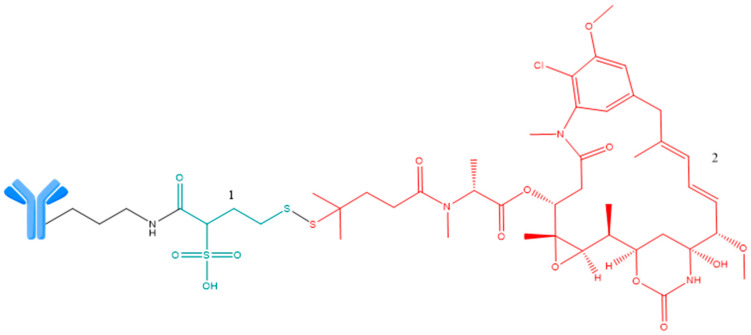
Mirvetuximab soravtansine ADC structure. 1—Cleavable Sulfo-SPDB linker (green); 2—payload (ravtansine, red). Adapted from [54].

A DAR of four has long been regarded as optimal, but recent studies have shown that this applies for ADCs with a second-generation linker that has DM or MMAE as a payload, such as mirvetuximab soravtansine (DAR 3.5) (Figure 13), anti-BCMA thiomab-amanitin (DAR 2), and depatuxizumab mafodotin (DAR 4) [54,87].

An example where a high DAR does not diminish the therapeutic efficacy of the active payload is seen in SG, an mAb anti-TROP-2 conjugated at SN-38 [54,88]. This ADC favors the release of large concentrations of SN-38 at the tumor level by attaching to a humanized IgG TROP-2 antibody, a surface glycoprotein that is expressed in over 90% of triple-negative breast cancer (TNBC) cases [89,90]. The success of this ADC is shown by its indication in TNBC, which had no treatment until the approval of SG by FDA in April 2020. A further unusual aspect of this ADC is the fact that the enhancement of the linker structure that includes a pegylated unit resulted in obtaining this ADC with a high DAR of 7.6 without diminishing its tolerance or efficiency [54].

Another example of ADC with high DAR is an exatecan derivative (DXd), a cytotoxic agent with an activity ten times more intense than the one of SN-38 in vitro, with an enhanced safety profile characterized by better solubility and with a bystander effect for the adjacent cancer cells, which is a benefit for heterogeneous tumors, and with a short half life to avoid off-target toxicity. The bioconjugation of DXd on trastuzumab anti-HER2 Cys residues through a proteolysis-sensitive maleimide linker made it possible to produce the fam-trastuzumab deruxtecan conjugate (DS-8201a) with a homogeneous DAR of 7.7 [54].

## 4. Development of ADCs

Strategies in the development of ADCs have evolved from first- to third-generation ADCs:

(i) The first generation is characterized by a conjugation of murine monoclonal antibodies and non-degradable linkers [91] that lack precise targeting to tumor tissue and high cytotoxicity [92,93].
(ii) The second-generation ADCs are characterized by some additional improvements, obtaining ADCs with enhanced target selectivity, using humanized antibodies with reduced immunogenicity, more efficient payloads, and stable linkers that have shown high effectiveness and clinical safety [94,95,96]. However, there are still drawbacks, including the existence of unconjugated antibodies and high DARs, leading to off-target toxicity, ADC aggregation, elevated metabolic degradation of the drug, and rapid clearance [94,97].
(iii) The third generation further refines previous shortcomings by employing specific conjugation optimization techniques, reducing the DAR to around 2–4 and reducing the proportion of unbound antibodies, thus enhancing the efficiency of ADCs [98]. Also, the fractional dosing regimen approach is a strategy that can extend the therapeutic index that may diminish the toxicity induced by the maximum concentration of ADCs in the systemic circulation, prolong the exposure time of ADCs in the tumor tissue, and maintain the intensity of the dose to assure antitumor efficacy [99].

### 4.1. First Generation ADC

The first generation of ADCs is represented by compounds where the selected payloads are cytotoxic molecules that act as DNA-disrupting agents (calicheamycin, SN-38, duocarmycin, doxorubicin) [29]. The payloads are conjugated to mAbs by monovalent, non-cleavable bonds or by acid-labile linkers. They are not entirely stable and have a narrow therapeutic index, but they represent an important first step in therapeutics in general [100]. Research conducted in the 1990s has resulted in the use of humanized mAbs in ADCs to achieve a more targeted action. This new ADC strategy has been affected by various drawbacks, ranging from unpleasant side effects to linker problems and challenges in delivering sufficient doses directly to the tumor site [101]. Thus, the initial strategies to create ADCs were mostly dominated by the following disadvantages [82,102,103,104,105].

(i) Drug efficacy—circulating serum concentrations were not in the therapeutic range: tumor cells express only a small quantity of antigen molecules, and the payload released by ADCs inside the cell is insufficient to meet the threshold concentration needed for cell destruction. Also, the quantity of ADC molecules delivered inside the cell is often lesser than the number of those that are attached to the cell surface;
(ii) Target antigen expression—they targeted receptors that lack sufficient selectivity for tumors, leading to unacceptable levels of toxicity;
(iii) Linker stability—the linkers employed were either excessively stable (leading to diminished potency and reduced efficacy) or excessively unstable (leading to compromised target specificity and heigh systemic toxicity). Thus, the short half life (43 h) of the hydrazone linker was a reason for unwanted toxicity because of early release of the drug off-target. The use of these first-generation linkers resulted in increased levels of payload dissociation still in circulation, leading to non-selective cytotoxicity. Linker instability remained an important issue in the 2000s, exemplified by GO, which released half of its payloads within 48 h into circulation. Because of this design flaw, GO was associated with increased mortality rate compared to other therapies;
(iv) Immune responses—the mAbs used in the first ADCs were either chimeric or murine, leading to an immune reaction and in the production of human anti-mouse antibodies which hindered the ability to administer repeated cycles of therapy.

Thus, critical concepts such as mAb immunogenicity, linker stability, and payload potency have emerged as extremely important directions drawn from first-generation and early second-generation ADCs [60].

GO with DAR 2-3 was approved by the FDA for use in CD-positive lymphoid leukemia, targeting CD33 [106,107]. This ADC is endocytosed by the target tumor cell, releases calicheamicin by hydrolyzing the linker, and acts by inducing breaks in double-stranded DNA, stopping the cell cycle and inducing apoptosis to the target tumor cell [108]. GO was later found to have no significant advantage compared to other classical cytotoxic drugs and to have severe hepatotoxicity [109], and it was voluntarily withdrawn from the market [110]. The potential therapeutic disadvantages of GO include the instability of the linker (it releases 50% of the active charge in about 48 h) and the fact that the active charge, calicheamicin, has high hydrophobicity, so that the proportion of binding with the monoclonal antibody is 50%, thus resulting in high toxicity [111]. Studies have also demonstrated that the gemtuzumab mAb can be removed from cells by the efflux pump [112].

In 2017, the second ADC of the first generation, inotuzumab ozogamicin, was approved. It is a humanized monoclonal antibody conjugated to calicheamicin through an acid-labile linker, indicated in acute lymphoblastic leukemia that expresses the CD22 marker [113]. This new ADC has the advantage of exhibiting fewer adverse effects than traditional cytotoxic chemotherapy [114]. 

### 4.2. Second Generation ADC: Site-Directed Conjugation

The second generation of ADCs was developed starting from the shortcomings and disadvantages of the first generation, especially as mAb technology experienced remarkable advancements, along with the development of mAbs isotypes, cytotoxic payloads, and also linkers [100,115,116]. mAbs were thus selected to enhance selective attaching to tumor cells while minimizing cross-reactivity with healthy cells, conjugating mAb IgG1 to small cytotoxic molecules with much higher selectivity than IgG4 [10]. To solve the problem of heterogeneity, research has focused on site-specific designed mAbs, including mAbs with modified Cys residues, amino acids from different sources, to make a more homogeneous product [37]. However, most second-generation ADCs have a narrow therapeutic window [117] due to a failure to reach the target [118], unconjugated antibody competition [119], and rapid aggregation or elimination of ADCs with a DAR from zero to eight. The mean DAR varies depending on the specific ADC: 3.5 for trastuzumab emtansine, 4 for BV, and 6 for inotuzumab ozogamicin [120,121]. In these ADCs, there is a low percentage of unconjugated mAb species of about 5% (e.g., BV and trastuzumab emtansine contain 5% unconjugated species), those that compete with drug-loaded species for attaching to the specific antigen, unlike GO with 50% non-conjugated species [70,94]. Furthermore, species with a DAR greater than four have been observed to generally exhibit lower tolerability, increased plasma clearance rates, and thus diminished in vivo effectiveness [121,122]. 

Other strategies that have contributed to increasing the efficiency and specificity of this generation of ADCs refer to the modulation of the hydrophobicity by using hydrophilic linkers and applying the chemical structure–therapeutic activity relationship. Higher-potency cytotoxic payloads such as auristatin and maytansine microtubule disruptors are used [37]. Multiple active cytotoxic molecules can be loaded onto each mAb without triggering antibody aggregation. Additionally, enhancements in both antibody carrier and cytotoxic payload, along with more efficient linkers in second-generation ADCs, contribute to achieving enhanced plasma stability and homogeneous distribution of DAR [46]. 

The linkers of this generation are characterized by a greater functionality than previous linkers, cleavable either enzymatically or by exposure to acid inside cells or lysosomes such as protease, hydrazine, polyethylene glycol (PEG), and disulfide linkers [48]. These linkers bring the advantage of releasing the payload at the targeted site and at the right time, also ensuring the stability of the ADC during preparation, storage, and systemic circulation [123].

With all the advantages of the second generation, there still are some aspects that require improvement: the insufficient therapeutic window because of off-target toxicity and the rapid aggregation or elimination of the payload from the increased DAR. When the DAR is above six, the ADC has increased hydrophobicity and its potency has the tendency to diminish because of faster in vivo distribution and clearance [124]. In view of this, optimizing DAR through specific conjugation coupled with ongoing refinements of linker, payloads, and mAbs may be the key to the successful advancement of the third Dgeneration of ADCs.

### 4.3. Third Generation ADCs

Research on third generation of ADCs explore a widening of the therapeutic window, with ways to increase their activity and specificity, such as developing of bispecific mAbs (both IgG-like and non-IgG-like) with two different binding sites [99]. For example, a single ADC can both release a toxin and activate natural killer cells through the bystander effect [125]. Another innovative technology is the use of antigen-binding fragments (Fabs) instead of intact mAbs [126]. These are sections of antibodies that include antigen-binding sites. These Fabs are highly stable, can be internalized more easily, are relatively easy to purify, and tend to have less immunogenicity [127,128]. The primary distinction between whole antibodies and antibody fragments lies in the molecular size, thus the 150 kDa IgG is diminished to 50 kDa in the case of Fabs, and even smaller sizes are observed for other antibody fragments [129]. The benefits of the development of site-specific conjugation technology were established by obtaining homogeneous ADCs with well-characterized DARs (two or four) and optimal cytotoxicity and better pharmacokinetic efficiency [123,130]. 

In 2019, the FDA approved polatuzumab vedotin, enfortumab vedotin, and fam-trastuzumab deruxtecan; in April 2020, sacituzumab govitecan was approved for TNBC. Two of the more recent ADCs (sacituzumab govitecan and loncatuximab tesirine) were developed with PEG chains incorporated into their linker technology to enhance in vivo solubility and stability [10].

The manufacturing technology of this generation of ADCs is focusing on therapeutic targeting. Thus, MEDI4276 (a biparatopic anti-HER2 mAb that targets two different non-overlapping epitopes on the same target, conjugated to four tubulysine moieties via a maleimidocaproyl linker), vadastuximab talirine (an anti-CD33 mAb conjugated to two PBD via a protease cleavable linker) and IMGN779 (anti-CD33 mAb conjugated to three indolinobenzodiazepine moieties via a cleavable disulfide linker) are examples of this technology [29]. 

Regarding the approval of third-generation ADCs, for liquid tumors, in 2000, GO was approved for acute myelogenous leukemia and was withdrawn from the market in 2010 and reapproved in 2017. BV was approved in 2011 for Hodgkin lymphoma, inotuzumab ozogamicin was approved in 2017 for acute lymphoblastic leukaemia, moxetumomab pasudotox was approved in 2018 for hairy cell leukaemia, and polatuzumab vedotin and loncastuximab tesirine were approved for beta-cell lymphoma in 2019 and 2021, respectively. The ADCs for solid tumors, trastuzumab etamsine and trastuzumab deruxtecan, were approved in 2013 and 2019, respectively, for HER2-positive breast cancer; enfortumab vedotin was approved for metastatic urothelial cancer in 2019; SG and cetuximab soratolacan were approved for triple-negative breast cancer and head and neck cancer, respectively, in 2020; in 2021, tisotumab vedotin and disitamab vedotin were approved for cervical cancer and advanced breast cancer, respectively; and in 2022, mirvetuximab soravtansine was approved for ovarian cancer [50,131]. 

## 5. Safety Issues

Despite their benefits, the formulation of ADCs has some safety concerns. One of the main problems is represented by off-target effects. These effects are produced in normal cells because of the interactions of cytotoxic drugs with molecular structures other than the target ones. Because of the lack of specificity for tumor cells, mAbs can interact in a small proportion with antigens expressed on healthy cells, leading to normal cell death by releasing the payloads in normal tissues [132]. On the other hand, the expression of specific antigens on the normal cells leads to off-target cytotoxic effects [133]. Another problem is represented by tumor heterogeneity. Tumor cells can express different levels of the target antigen. Some of them present a high level of antigen, others a low level of antigen, and others can be negative. Combined with the antigen expression in normal cells, this heterogeneity determines the drug resistance and implicitly the disease relapse [134]. During the systemic circulation of ADCs administered by the intravenous route, the cytotoxic drug can have cytotoxicity in the normal tissue by binding to other off-target antigens [135]. The premature release of the payloads could be determined by linker stability. The consequence of this mechanism is represented by off-target effects in normal tissues, too [39]. Also, the immune system can interact with the ADC (e.g., immune cells expressing Fc receptors) and consecutively lead to its elimination; it can also activate the immune response against normal cells expressing the target antigen [135]. Many strategies were proposed to mitigate the cytotoxicity of ADC: (i) the optimization of the antibody specificity to target antigens; (ii) the judicious selection of target antigens expressed on normal cells in a very low proportion, but very expressed on tumor cells; (iii) the use of stable linkers that permit the release of payloads on-target; (iv) the preclinical evaluation of off-target toxicity; (v) the patients’ careful monitoring during clinical trials to manage adverse reactions [135,136].

On the other hand, the use of payloads with small differences between their therapeutic and toxic doses is another factor that contributes to off-target effects [121]. Moreover, the quick clearance and inefficient metabolism of ADCs can determine systemic toxicity [94,136,137], including toxic effects on organs such as the liver or kidneys [138,139]. The neurologic effects are another limitation of ADC use, especially the payloads targeting tumors of the central nervous system [139]. In some cases, an immune response against ADCs components (mAbs, payload) could be triggered. Therefore, the immunogenicity of ADCs can be mitigated by using of human or humanized antibodies [140]. 

Lastly, the toxicity of ADCs may be the result of interactions with other medications. First, ADCs may interact with other cytotoxic drugs. An example is represented by the association between trastuzumab emtansine and pertuzumab even with a low risk of pharmacokinetic interactions [141]. Other interactions alter the metabolism of the ADC. Thus, different enzyme inhibitors or inducers could target the same enzymatic substrate like the payloads. For example, GO is mainly metabolized by CYP1A2, CYP3A4, CYP2C8, etc.; BV is metabolized by CYP3A4; trastuzumab emtansine is metabolized by CYP3A4/5; inotuzumab ozogamicin is metabolized by CYP or UGT, etc. [138,142].

## 6. Clinical Trials

Since the year 2000, several ADCs have been approved by the FDA and EMA for various hematological and solid neoplasms. The research regarding these molecules is in continuous development, and many clinical trials are underway. Thus, the interest of the scientific and medical community, alongside the high expectation of ADC effectiveness, is proven by the large number of clinical trials (215 on the 19 February 2024) enlisted by the ClinicalTrials.gov database. A total of 36 clinical trials were not yet recruiting patients at that time. Table 3 presents some of the most promising molecules that have reached advanced stages in phase 3 or phase 2/phase 3 clinical trials until 19 February 2024 [143].

## 7. Perspectives and Challenges 

The effectiveness of conventional antitumor therapies, starting from classical, non-specific chemotherapy to molecular targeted therapy, encounters some issues due to the high toxicity of classical chemotherapy and to the inadequate cytotoxicity and labelling ability of target genes for molecular targeted therapy [144,145].

Since the initial introduction of ADCs in therapeutics, numerous advantages over classical chemotherapy have emerged. These include the development of compounds with increasingly selective cytotoxicity against tumor cells, avoiding healthy cells, enhancing of the therapeutic index and therapeutic window, overcoming the low selectivity, and rapid clearance associated with chemotherapy. Additionally, they address the suboptimal antitumor efficacy of targeted therapy [31,146].

Despite these advantages, there are still many challenges in the development of ADCs and in elaborating strategies that lead to the improvement of current treatments, such as selecting specific antigens as targets and addressing structural optimization strategies [147]. 

### 7.1. Selection of Specific Antigens

Restrictive targeting strategies for extracellular antigens with highly homogeneous expression on tumors but reduced expression in healthy tissues can enhance the selectivity of targeted ADCs and thus lead to decreased toxicity of the active load [148]. It is important to consider that solid tumor antigens are highly heterogeneous and dynamic, so that the range of antigen selection can be extended to negative antigens or those that cannot sufficiently induce tumor internalization while also taking into account the bystander effect [149,150].

Mutant oncogene targets can also be included as potential antigens, especially highly and homogeneously expressed mutant oncogenes with high internalization possibilities [151,152]. Oncogenic antigens may optimize ADC action indirectly by down-regulating expression causing tumor resistance and for further antitumor effects that arise via antibody-mediated inhibition of downstream signaling pathways [153,154].

### 7.2. Structural Optimization of ADCs

Antibodies are a group of biomolecules widely used in therapeutics and diagnostics that require modifications with specific functional fragments, including fluorophores, active cargos, and proteins. Several methods in ADC technology involve the stable attachment of active cargos to lysine or Cys residues in the mAb that inevitably yield heterogeneous products that cannot be further purified [155]. Thus, special technologies are employed to obtain homogeneous antibody conjugates by bioorthogonal handles specifically introduced by different enzymatic approaches, genetic code extension, or genetically encoded tagging. Subsequently, functionalization is carried out using bioorthogonal conjugation reactions [156]. The resulting homogeneous products have been shown to be superior to heterogeneous counterparts for both in vitro and in vivo settings [157].

There is a continuous interest in developing site-specific labeling strategies for antibodies, a concern arising from the mAb advantages of high therapeutic indices and the remarkable biochemical properties of antibody conjugates specific to a particular therapeutic target. Consequently, a range of methods have been developed for the specific covalent attachment of functional molecules to antibodies. Most of these techniques need specific antibody engineering approaches to insert a distinct reactive fragment, which is selectively modified by using bioorthogonal chemistry. In this strategy for accurate labeling of native antibodies, the capacity to selectively modify a single amino acid residue with increased efficiency and no off-target effects is essential [158,159]. Thus, for example, a new antibody manufacturing process has been developed by metabolic engineering of Cys, tested on laboratory animals in Chinese hamster ovary cells, employing a specific Cys coating technology. This technology facilitates direct conjugation of the active cargo subsequent to chemoselective reduction with tris(3-sulfonatophenyl)phosphine, with the intent to merge Cys-based site-specific conjugation with other site-specific conjugation methods to research ADCs and capitalize on multiple mechanisms of action for efficacious cancer treatments [160].

Consequently, in order to meet all criteria in the development of an ideal ADC, the following aspects must be considered: (i) the technology should be applicable to any mAb without the need for prior purification and/or chemical modification and (ii) the labeling procedure should not alter the attachment properties of the mAb [161]. Most ADCs developed until now have been obtained using random conjugation to lysine or Cys residues. Nevertheless, since antibodies have about 40 lysine residues exposed on their surface and several Cys residues, payload binding generally results in a very heterogeneous mixture [162]. Random conjugation can alter the efficacy, safety, pharmacokinetics, and immunogenicity of the conjugated antibody, so strategies are needed to direct the development of means of attaching the payload to a specific antibody site [75].

The regioselectivity of these chemical modifications ought to be governed by the labeling fragment rather than by engineering or chemical modification of the antibody itself; thus, by modeling the chemical reaction with an aptamer [163], a small molecule [164], a protein [165], or a peptide [166], it has been possible to label unmodified proteins. Studies have been carried out on the approach of new metal-mediated cleavable linkers with single-caged fragments that are designed for well-controlled payload release by the bioorthogonal bond cleavage reaction and toxicity diminution by substoichiometric amounts of metals that can reach the desired effectiveness [167,168]. Metal-mediated linkers are based on palladium, ruthenium, copper, and platinum [169]. 

On the other hand, the conjugation strategy approached in the constitution of an ADC alters the homogeneity of the DAR, the release of cytotoxic payloads, and toxicity outside the therapeutic target. Given the inefficient chemistry and immunogenicity, novel technologies are currently being researched for better-controlled DAR and ADC homogeneity. Thus, for instance, using the dolaflexin platform, a novel ADC, XMT-1536, targeting sodium phosphate cotransporter protein type II (NaPi2b), connected by a water-soluble polymer to enhance pharmacokinetics, solubility but also immunogenicity with a DAR of 10-12 is being evaluated [170,171].

### 7.3. Strategies to Optimize Resistance and Toxicity

The development of tumor resistance to drugs is still a major challenge as it can often occur without an explicit mechanism. Current formulated hypotheses regarding resistance mechanisms are (i) changes in the tumor microenvironment leading to decreased penetration of the drug agent into the tumor cell, (ii) some deficiencies in the internalization pathways adapting resistance to payloads, (iii) down-regulation of antigens [92,172], and (iv) induction of the active efflux of cytotoxic payloads that are transported by ATP-binding cassette transporter proteins, like multidrug resistance protein 1 [173]. To improve the effectiveness of ADCs, some studies aim to circumvent or even block some resistance mechanisms [94]. On the other hand, toxicity is an important factor limiting the clinical use of ADCs, depending on the physiological role of antigens in non-tumor tissues, linker stability, quantity and characteristics of payloads, and the bystander effect [174,175,176]. Adverse effects of ADCs are important and must be carefully monitored, steadily prevented, and treated in a timely manner with appropriate modifications to dosing regimens during clinical use. Both optimizing the structure of ADCs and adjusting dosing regimens may serve as potential solutions to reduce toxicity [95]. 

## 8. Conclusions

The introduction of ADCs into therapeutics in the management of neoplastic diseases has been a highly important advance in the treatment of tumor refractory to classical therapies and personalized medicine. Through research on ADCs, the therapeutic index and indications are expanded, and limitations due to heterogeneously expressed antigens are overcome by the bystander effect and non-interfering mechanisms. There is also a broad horizon for exploration of early-stage neoplasms along with combination therapy approaches. Further, the mechanisms of emergence of tumor drug resistance, optimizing treatment strategies, and appropriate patient personalization are still topics of major importance, along with techniques to obtain improved ADCs.

## Figures and Tables

**Table 1:** Classification of linkers in ADCs.

Linker Type	Linker Cleavage Mechanism	Chemistry of Linker
Cleavable	Chemically cleavable	pH sensitive
		Reduction-sensitive
	Enzymatically cleavable	Peptide-based
		β-glucuronide based (β-glucuronidase and β-galactosidase sensitive)
		Phosphate based
Non-cleavable	N.A. *	Thioether
		Maleimido caproyl

* N.A.—not applicable.

**Table 2:** The effects resulting from different strategies of conjugation [75,85,86].

Conjugation Strategy	Effects	
Increasing DAR	+	increased potency
	−	increased protein aggregation
	−	altered biological functions
	−	induced immune responses by breaking B-cell tolerance
	−	increased antibody clearance
	−	reduced exposure to drug
	−	increased toxicity
	−	increased protein aggregation
Drug attachment by proximal sites to the antigen-binding region	−	in vivo and in vitro conjugate stability affected
	−	linker instability
	−	drug exposure
Symmetric attachment of DAR by antigen-binding region	+	increased efficacy
	+	increased tolerability

+ beneficial outcome; − non-beneficial outcome.

**Table 3:** Examples of ongoing phase 3 clinical trials on ADCs [143].

Conditions	ADC	Number of Enrolled Patients	Last Update
Breast cancer	Dato-DXd	1728	5 February 2024
Breast cancer	Trastuzumab deruxtecan	250	5 January 2024
Acute myeloid leukemia	Gemtuzumab ozogamicin	700	10 August 2021
Breast cancer	Dato-DXd	600	17 January 2024
Breast cancer	Dato-DXd	1075	13 February 2024
Solid cancer	R-DXd	650	14 December 2023
Squamous cell carcinoma of the head and neck	MRG003	180	2 March 2023
Epithelial ovarian cancer/peritoneal cancer/ fallopian tube cancer	Mirvetuximab Soravtansine	35	21 November 2022
Breast cancer	Recombinant Anti-HER2 Humanized Monoclonal Antibody—Monomethyl Auristatin F Conjugates for Injection (FS-1502)	314	1 September 2023
NSCLC	Datopotamab deruxtecan	1280	6 February 2024
Breast cancer	Dato-DXd	625	24 January 2024
Breast cancer	RC48	366	24 January 2024
Ovarian cancer/peritoneal cancer/ fallopian tube cancer	Mirvetuximab soravtansine plus bevacizumab	418	8 February 2024
Multiple myeloma	Belantamab mafodotin	357	2 November 2023
Advanced breast cancer/metastatic breast cancer	MRG002 Trastuzumab Emtansine for Injection	350	17 March 2023
Endometrial cancer	MK-2870	710	19 January 2024
B-cell non-Hodgkin lymphoma	Loncastuximab tesirine	210	14 August 2023
HER2-negative breast cancer/triple-negative breast cancer	Sacituzumab govitecan	1332	2 March 2023
Small-cell lung cancer	Ifinatamab deruxtecan	468	12 January 2024
Lugano classification limited stage Hodgkin lymphoma AJCC v8	Brentuximab vedotin	1875	16 February 2024
Carcinoma, non-small-cell lung	Sacituzumab govitecan	614	9 February 2024
Advanced or metastatic urothelium cancer	MRG002	290	13 April 2023
Acute myeloid leukemia	Gemtuzumab ozogamicin	1400	22 January 2024
NSCLC	MK-2870	556	19 January 2024

NSCLC—Non-small Cell Lung Cancer, Dato-DXd—Datopotamab deruxtecan, R-DXd—Raludotatug deruxtecan, MRG003—Anti–epidermal growth factor receptor antibody drug conjugate, RC48—Disitamab vedotin, MRG002—HER2-targeted antibody-drug conjugate, MK-2870—Sacituzumab tirumotecan.

## Data Availability

Data are contained within the article.

## Abbreviations

ADC	Antibody–drug conjugate
Ala	Alanine
BV	Brentuximab vedotin
Cit	Citrulline
Cys	Cysteine
DAR	Drug–Antibody Ratio
Dato-DXd	Datopotamab deruxtecan
DM1	Mertansine/emtansine (maytansinoid)
DM4	Soravtansine/ravtansine (maytansinoid)
DOX	Doxorubicin
DS-8201a	Trastuzumab deruxtecan
DXd	Exatecan derivative
EMA	European Medicines Agency
Fabs	Antigen-binding fragments
Fc	Fragment crystallizable region
FDA	Food and Drug Administration
G2	Second growth phase
GO	Gemtuzumab ozogamicin
HER2	Human epidermal growth factor receptor 2
huC242-SPDB-DM4	Cantuzumab ravtansine
Le(Y)	Anti-Lewis Y
Lys	Lysine
M	Mitosis
mAb	Monoclonal antibody
MC	Maleimidocaproyl
MK-2870	Sacituzumab tirumotecan
MMAE	Monomethyl auristatin E
MRG002	Human Epidermal Growth Factor Receptor 2 Positive Breast Cancer
MRG003	Anti-Epidermal Growth Factor Receptor Antibody Drug Conjugate
NSCLC	Non-small cell lung cancer
PABC	P-aminobenzyloxycarbonyl
PBD	Pyrrolobenzodiazepine dimer
PEG	Polyethylene glycol
Phe	Phenylalanine
PK	Pharmacokinetics
RC48	Disitamab vedotin
R-DXd	Raludotatug deruxtecan
SG	Sacituzumab govitecan
SN-38	Active metabolite of iridotecan
SSC	Site-specific conjugation
T-DM1	Trastuzumab emtansine conjugate DM1
TNBC	Triple-negative breast cancer
Val	Valine
